# Induction of Mutations in *Veronica* Species by Colchicine Treatment

**DOI:** 10.3390/life15091367

**Published:** 2025-08-28

**Authors:** Hye-Wan Park, Samantha Serafin Sevilleno, Ji-Hun Yi, Wonwoo Cho, Young-Jae Kim, Yoon-Jung Hwang

**Affiliations:** 1 Department of Convergence Science, Sahmyook University, Seoul 01795, Republic of Korea; hyewan00@korea.kr; 2 Gardens and Education Division, Korea National Arboretum, Pocheon 11186, Republic of Korea; kimla9833@korea.kr; 3 Plant Genetics and Breeding Institute, Sahmyook University, Seoul 01795, Republic of Korea; samanthasevilleno@syuin.ac.kr; 4 Forest Biological Resources Utilization Center, Korea National Arboretum, Yangpyeong 12519, Republic of Korea; easy2641@korea.kr (J.-H.Y.); wonwoocho85@korea.kr (W.C.)

**Keywords:** colchicine, mutation breeding, phenotype, ploidy level, *Veronica*

## Abstract

*Veronica nakaiana* Ohwi and *Veronica pusanensis* Y.N.Lee are rare and endemic plants native to Korea, with increasing interest in their cultivation and breeding for industrial applications. Mutation breeding is important for developing horticultural cultivars. Among mutation breeding techniques, chemical mutagenesis is particularly accessible and effective. Colchicine-induced mutagenesis was performed in vivo at various concentrations (0.2%, 0.4%, 0.6%, 0.8%, and 1.0%) and treatment durations (1, 2, 3, 4, and 5 h). Both *V. nakaiana* Ohwi and *V. pusanensis* Y.N.Lee showed the highest survival (23.4% and 34.8%, respectively) and mutation (1.6% and 0.5%, respectively) rates with 0.2% colchicine. Flow cytometry and chromosome number analyses revealed mutants as tetraploid, with chromosome numbers ranging from 2*n* = 66 to 2*n* = 68. Stomatal analysis indicated increased stomatal length and width and decreased stomatal density. Morphological analysis of the mutants revealed that the leaves of *V. nakaiana* Ohwi and *V. pusanensis* Y.N.Lee were significantly larger and had different shapes compared to the control. This study successfully generated new mutant plants of two *Veronica* species using chemical mutagen treatment, which could be utilized as new genetic resources for various *Veronica* species breeding programs in the future.

## 1. Introduction

*Veronica* L., commonly called speedwell [1], was formerly assigned to the family Scrophulariaceae, but has been reclassified into the family Plantaginaceae in recent studies [2]. *Veronica* comprises nearly 450 species worldwide [3], including approximately 180 species in the Southern Hemisphere and more than 250 species of the annual and perennial Hebe complex in the Northern Hemisphere [4,5]. *Veronica* species appear in a wide range of ecological habitats, from aquatic to semi-desert [6], and are distributed geographically throughout the Northern Hemisphere and Australia (Australia, New Zealand, and New Guinea), with centers of diversity identified in New Zealand and Western Asia [7]. Flowers have a long flowering period from spring to summer, come in a variety of colors, including white, pink, and purple, and have high horticultural value [1].

In Korea, 25 species have been reported, including endemic plants, such as *Veronica ovata* Nakai, *Veronica nakaiana* Ohwi, *Veronica pusanensis* Y.N.Lee, and *Veronica kiusiana* var. *diamantiaca* (Nakai) T.Yamaz [8]. *V. nakaiana* Ohwi is endemic to Ulleungdo Island [9] and has been designated as an endangered species by the International Union for Conservation of Nature (IUCN) [10]. *V. pusanensis* Y.N.Lee is a rare species that requires protective measures. It can be found in small colonies between rocks or on slopes distributed only in the coastal areas of Busan, South Korea [11].

To industrialize indigenous *Veronica*, studies have been conducted on the growth characteristics of *Veronica* seeds [12] and their cultivation and propagation methods [13,14]; however, the available information remains limited. Despite this situation, indigenous plants distributed in the domestic flower market accounted for only 14% of the total in 2017 [15]. A need has risen to develop and advance new breeding technologies that can replace and compete with foreign breeds [16].

Mutation breeding, a method proposed to overcome these limitations, is a technology that induces rapid genetic changes in organisms by introducing mutations through biological, chemical, and physical factors, rather than through genetic recombination or isolation [17,18]. It can introduce new traits by modifying existing phenotypic characteristics, such as the color, shape, and pattern of the leaves and flowers of plants, improving the desirability of the plant and increasing its marketability [19]. Physical mutagens such as gamma rays, fast neutrons, and ionizing radiation can generate considerable genetic diversity, but can be detrimental by causing chromosomal breakage [17,20]. In contrast, chemical mutagens are preferred because they are easy to handle and use, and do not require sophisticated facilities or equipment [21]. Colchicine induces polyploidy in plants primarily by inhibiting the formation of microtubules that separate chromosomes [22]. This effect increases cell size, allowing the production of larger organs (i.e., leaves, branches, flowers, and fruits) [23,24].

This study aimed to induce polyploidy in two endemic *Veronica* species by treating them with colchicine, a chemical mutagen, at various concentrations and durations. In addition, (1) the survival and mutation rates for each treatment group were determined, (2) cytogenetic studies were performed to select putative mutants, and (3) stomatal characteristics and morphological analyses of the leaves of mutant plants were performed.

## 2. Materials and Methods

### 2.1. Plant Materials

Seeds of *V. nakaiana* Ohwi (IT 231002) and *V. pusanensis* Y.N.Lee (IT 231003), two species of *Veronica* taxa native to Korea, were obtained from the Useful Plant Resources Center of the Korea National Arboretum, Republic of Korea. The seeds were germinated in a moistened filter paper at room temperature (RT) until the radicles grew to approximately 1.5–2 cm for use in mutation induction.

### 2.2. Mutation Induction by Chemical Mutagens

In this study, a completely randomized design using a 5 × 5 factorial arrangement was set up with 25 treatment combinations of five colchicine concentrations (0.2%, 0.4%, 0.6%, 0.8%, and 1.0%) and five treatment times (1, 2, 3, 4, and 5 h). For each treatment group, 100 seeds were soaked. The control group contained untreated seeds. The colchicine concentrations (0.2–1.0%) and treatment durations (1–5 h) were selected based on ranges commonly reported in mutation breeding studies of ornamental and medicinal plants [25]. These ranges are generally sufficient to induce chromosome-doubling while minimizing lethal effects. We included lower concentrations and shorter durations to reduce mortality, while also testing higher doses to assess toxicity thresholds.

A 1% colchicine solution (Sigma-Aldrich Corp., Yongin, Republic of Korea) was prepared and diluted with distilled water according to the treatment concentration. According to the safety protocol, plants added to the colchicine solution (200 μL) were stored in a dark hood to prevent light exposure during the treatment process. After the specified treatment time, the plants were washed with distilled water and planted in pots containing horticultural substrates (Baroker, Seoul Bio Co., Ltd., Chungbuk, Republic of Korea).

### 2.3. Flow Cytometry (FCM) Analysis

An FCM analysis for DNA content determination was performed based on the method described by Doležel et al. [26]. Approximately 20 mg of young, healthy leaves were placed in a Petri dish containing 800 μL of ice-cold LB01 lysis buffer for nuclei isolation and minced with a sharp razor blade. The mixture was filtered twice through a 50-mm nylon mesh (Shanghai Bolting Cloth Manufacturing Co., Ltd., Shanghai, China) and stained by simultaneously adding 50 μL of propidium iodide (Sigma-Aldrich, St. Louis, MO, USA, Cat. No. P4170; Molecular Probes; Cat. No. P3566) and 3 μL of RNase (Sigma-Aldrich, St. Louis, MO, USA, Cat. No. R5000).

In total, 300 μL of each sample was transferred to a cell culture plate, and the fluorescence peaks of the standards and samples were analyzed using CytExpert v2.3 software (Beckman Coulter Inc., Pasadena, CA, USA) on a CytoFLEX flow cytometer equipped with a 50 mW, 488 nm solid-state diode laser (Beckman Coulter Inc., Pasadena, CA, USA). Each sample was measured at least 2000 times, and the measurements were repeated at least three times to obtain the mean and standard deviation.

### 2.4. Chromosome Preparation

Young root tips from the control (diploid) and plants with confirmed polyploidy (tetraploid) by flow cytometry analysis were collected in the morning when cell division was most active. The roots were pretreated with 2 mM 8-hydroxyquinoline and stored at 18 °C for 5 h. Roots were fixed overnight in Carnoy’s solution (3:1, ethanol/acetic acid, *v*/*v*) at 25 °C, transferred to 70% ethanol solution and stored at 4 °C. The roots were washed with distilled water and treated with 60 µL enzyme solution (1% cellulose, cytohelicase, and pectolyase) at 37 °C for 90 min. The enzyme-treated roots were transferred to a 1.5 mL tube containing Carnoy’s solution and homogenized.

The homogenized root meristem was placed on ice for 5 min and centrifuged at 13,000 rpm for 4 min. Following centrifugation, the supernatant was discarded, and the pellet was immediately resuspended in an acetic acid–ethanol (9:1, *v*/*v*) solution. The final suspension was applied to a glass slide preheated to 80 °C in a humid chamber and air-dried at RT.

The prepared chromosome slides were counterstained with 1 μg·mL^−1^ of 4′, 6-diamidino-2-phenylindole (DAPI) (Roche, Indianapolis, IN, USA) and observed under an Olympus BX53 fluorescence microscope (Olympus, Tokyo, Japan) with a built-in a Charge Coupled Device (CCD) camera (CoolSNAP™ cf, Photometrics, Tucson, AZ, USA) using an oil lens.

### 2.5. Preparation for Fixation Methods

Fixation methods for the observation of leaf stomata were performed with some modifications from the method of Ha et al. [27]. The mid-green part of the central lamina of each mature leaf of a diploid control and tetraploid plant, as confirmed by chromosome number counting, was collected and used for analysis. The leaves were soaked in a fixative prepared using a formaldehyde-acetic acid-alcohol (FAA) solution (10 mL 95% ethyl alcohol, 5 mL glacial acetic acid, 50 mL formaldehyde, 35 mL and distilled water) at RT for 90 min and then rinsed with distilled water. As the samples were observed under a scanning electron microscope (SEM), the xylene series was omitted and the samples were dehydrated in an ethanol series (50%, 70%, and 100%) for 30 min each. The final samples were stored in 70% ethanol at 4 °C until further use in the next experiment.

### 2.6. Stomata Characteristics

All samples were dried at 10 °C for 5 min using a critical point dryer (CPD) (SPI-DRY Critical Point Dryer, Regular, Structure Probe, Inc., West Chester, PA, USA) and then coated with gold using a coating device (NeoCoater, MP-19020NCTR, JEOL Ltd., Tokyo, Japan). The microstructure of the abaxial leaf surface was observed using an SEM (JSM-6510, JEOL Ltd., Tokyo, Japan) at an accelerating voltage of 10–15 kV.

### 2.7. Leaf Morphological Evaluation

In this study, leaf characteristics of *Veronica* mutants and control plants older than one year were investigated to determine the morphological stability of polyploid plants. Morphological data were collected to evaluate the polyploidy of diploids and selected polyploids *V. nakaiana* Ohwi and *V. pusanensis* Y.N.Lee. Leaf characteristics were examined using a stereoscopic microscope (Olympus SZ61, Olympus, Tokyo, Japan) equipped with ToupView software (v. 3.7, ToupTek Photonics, Hangzhou, China). The largest leaves on the plant were selected and data on the characteristics of the leaf tip, margin, and petiole were collected to compare the shapes of each.

### 2.8. Data Collection and Analysis

The plants treated with colchicine were collected two weeks after replanting into plug trays, and the survival rate was calculated by dividing the total number of surviving plants in the treatment groups by 100. The number of surviving mutant plants was determined and confirmed using flow cytometry and chromosome analyses.

To determine stomatal density, six counts were conducted per leaf at random locations across the entire leaf surface observed at ×400 magnification. Stomatal length and diameter were measured using ImageJ (v. 1.52a, National Institutes of Health, Bethesda, MD, USA), and the average length was calculated from ten images.

For morphological measurements, data was collected by measuring the length, width, and area of leaves using ImageJ software (v. 1.52a, USA). Numerical data obtained from morphological analyses were subjected to analysis of variance (ANOVA) using SPSS (version 20, IBM Corp., Armonk, NY, USA). Significant differences between means were analyzed using Duncan’s multiple range test (DMRT) at a 5% significance level.

## 3. Results

### 3.1. Survival and Mutation Rate

*V. nakaiana* Ohwi and *V. pusanensis* Y.N.Lee with their survival and mutation rates in response to mutagen treatment are presented in Table 1.

The survival rate of *V. nakaiana* Ohwi was 15.9%, with 397 individuals remaining. The highest survival rate (117 individuals) was observed at 0.2% concentration among the treatment groups. As the concentration of the mutagen increased, the survival rate decreased, with the lowest survival rate recorded at 1.0% concentration, wherein only 52 individuals survived. FCM analysis identified 27 mutants, corresponding to a mutation rate of 6.8%. Mutants were detected across all treatment concentrations, with one or two mutants identified per group. Notably, the T8 group (0.4% colchicine for 3 h) exhibited the highest number of mutants, with three individuals (Table 1).

*V. pusanensis* Y.N.Lee showed a survival rate of 23.8% with 596 surviving individuals. Additionally, nine mutants showed a mutation rate of 1.5%. The survival rate of *V. pusanensis* was higher than that of *V. nakaiana* Ohwi at all concentrations. Similar survival rates were observed at all concentrations, except for 0.8%. Mutations occurred at all concentrations except 0.8%, and the mutation rate was significantly lower than that of *V. nakaiana* Ohwi. *V. pusanensis* Y.N.Lee produced one mutant in each of the treatment groups at all time points except for the 4 h treatment group at the lowest concentration of 0.2%, and two mutants were observed in each of the 1 h treatment groups at 0.4% and 0.6%. Despite high survival and mutation rates at low concentrations, only one mutant [T23 (1.0% + 3 h)] was confirmed (Table 1).

### 3.2. Nuclear DNA Genome Size Estimation

**Figure 1:**
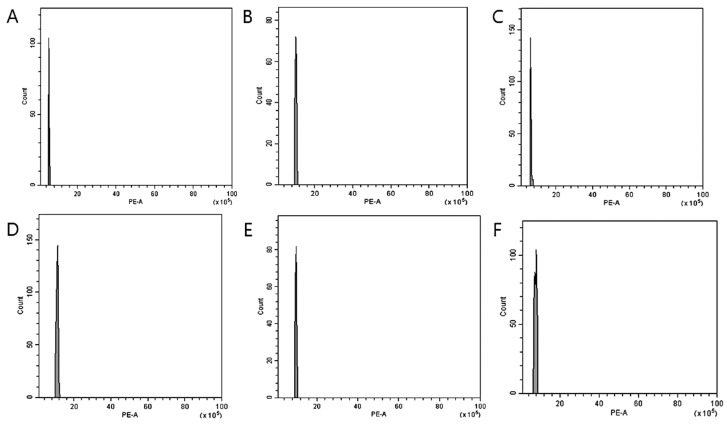
Estimation of the nuclear DNA content of *V. nakaiana* Ohwi following colchicine treatment by flow cytometry: (**A**) control, (**B**) 0.4% + 3 h (T8-P1); (**C**) 0.4% + 3 h (T8-P2); (**D**) 0.4% + 3 h (T8-P3); (**E**) 0.6% + 1 h (T11-P5); and (**F**) 1.0% + 3 h (T23-P2), where (**B**–**F**) show equivalent peaks (4*x*) compared to the control (2*x*). The X-axis indicates the PE-A or propidium iodide fluorescence intensity, while the Y-axis of each figure indicates the count of nuclei.

**Figure 2:**
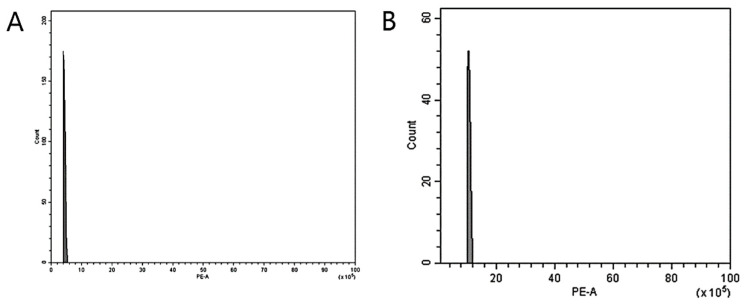
Estimation of the nuclear DNA content of *V. pusanensis* Y.N.Lee following colchicine treatment by flow cytometry: (**A**) control; and (**B**) 0.6% + 1 h (T11-P4), where (**B**) show equivalent peaks (4*x*) compared to the control (2*x*). The X-axis indicates the PE-A or propidium iodide fluorescence intensity, while the Y-axis of each figure indicates the count of nuclei.

FCM was used to analyze the ploidy levels to confirm the induction of mutations in two *Veronica* species following colchicine treatment. The histogram illustrates the 2C peaks of the diploid control plants of *V. nakaiana* Ohwi and *V. pusanensis* Y.N.Lee found between channels 2 and 4 (Figure 1A) and channel 4 (Figure 2A), respectively. The identified mutants of *V. nakaiana* Ohwi (Figure 1B–F) and *V. pusanensis* Y.N.Lee (Figure 2B) showed 2C peaks in channel 8, indicating a significant peak difference in genome size estimates compared to the control plants.

### 3.3. Chromosome Number Confirmation

In *Veronica*, only mutants that produced young roots were selected for chromosome number analysis. In this study, chromosomes were counted in the control and mutant plants of *V. nakaiana* Ohwi and *V. pusanensis* Y.N.Lee (Table 2).

**Figure 3:**
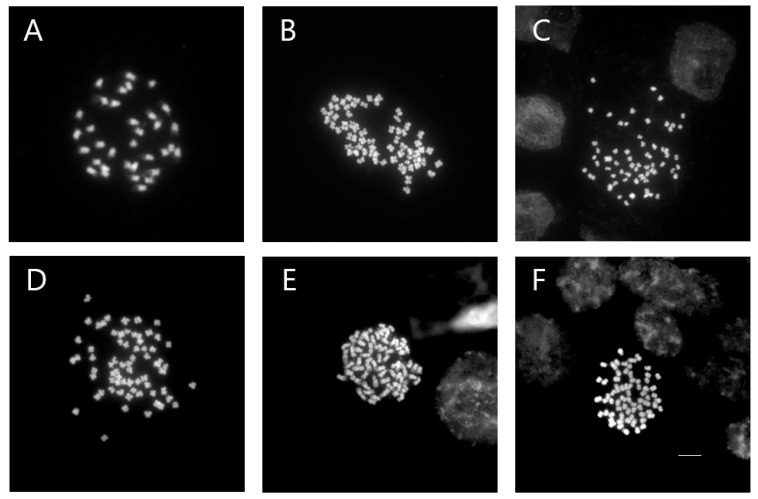
Mitotic chromosomes of *V. nakaiana* Ohwi control (**A**) and mutants (**B**–**F**) using DAPI staining: (**A**) control, (**B**) T8-P1 (0.4% + 3 h), (**C**) T8-P2 (0.4% + 3 h), (**D**) T8-P3 (0.4% + 3 h), (**E**) T11-P5 (0.6% + 1 h), and (**F**) T23-P2 (1.0% + 3 h). Scale bars = 10 μm, 400× magnification.

**Figure 4:**
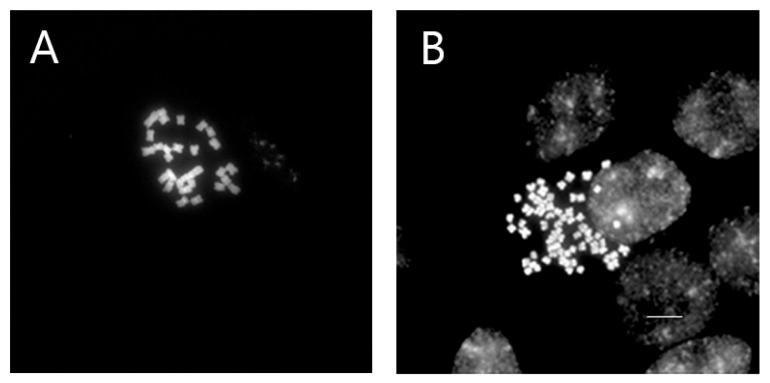
Mitotic chromosomes of *V. pusanensis* Y.N.Lee control (**A**) and mutant (**B**) using DAPI staining: (**A**) control, and (**B**) T11-P4 (0.6% + 1 h). Scale bars = 10 μm, 400× magnification.

Whereas all control plants had 2*n* = 2*x* = 34, the chromosome numbers of colchicine-treated *V. nakaiana* Ohwi (2*n* = 67 or 68) and *V. pusanensis* Y.N.Lee (2*n* = 66) doubled, confirming tetraploidy (2*n* = 4*x* = 68) (Figure 3 and Figure 4).

In *V. nakaiana* Ohwi, 2*n* = 68 chromosomes were observed in two mutants of the T8 (0.4% + 3 h) treatment group, as well as in T11-P5 (0.6% + 1 h) and T23-P2 (1.0% + 3 h); however, one mutant plant from T8 exhibited 2*n* = 67. In contrast, T11-P4 (0.6% + 1 h) of *V. pusanensis* had 2*n* = 66 chromosomes.

### 3.4. Evaluation of Stomatal Characteristics

In the present study, we compared the length, diameter, and density of stomatal cells between diploid control and tetraploid plants (Table 3).

**Figure 5:**
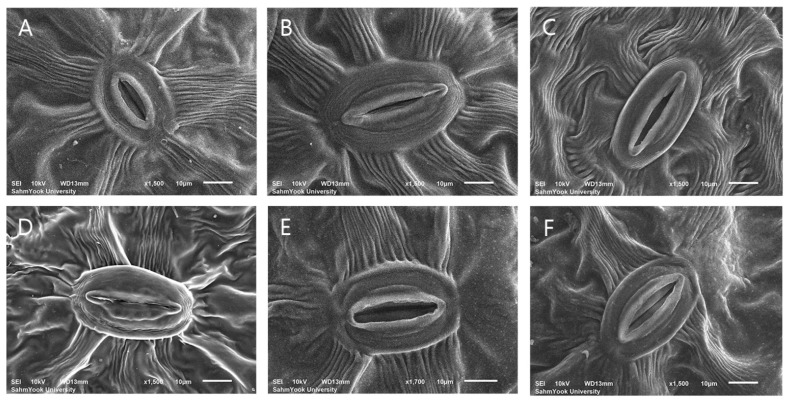
Comparison of stomatal sizes among colchicine-induced *V. nakaiana* Ohwi observed using scanning electron microscopy (SEM): (**A**) control, (**B**) T8-P1 (0.4% + 3 h), (**C**) T8-P2 (0.4% + 3 h), (**D**) T8-P3 (0.4% + 3 h), (**E**) T11-P5 (0.6% + 1 h), and (**F**) T23-P2 (1.0% + 3 h). Scale bars = 10 μm.

**Figure 6:**
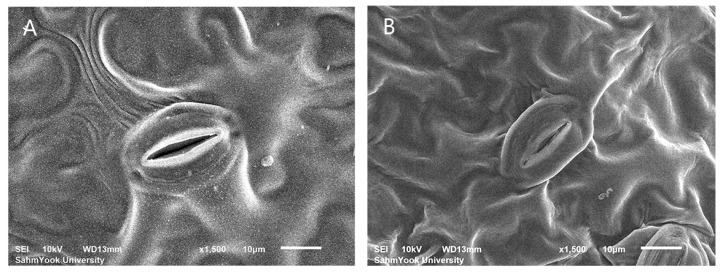
Comparison of stomatal sizes among colchicine-induced *V. pusanensis* Y.N.Lee observed using scanning electron microscopy (SEM): (**A**) control, and (**B**) T11-P4 (0.6% + 1 h). Scale bars = 10 μm.

According to the results, the use of the colchicine mutagen had different effects on the changes in stomatal cell size of *Veronica* in the two species (Figure 5 and Figure 6).

The average stomatal cell length of *V. nakaiana* Ohwi diploid plants was 30.59 ± 1.52 μm, which increased to 46.53 ± 2.90 μm (T8-P1), 34.30 ± 3.88 μm (T8-P2), 36.14 ± 3.12 μm (T8-P3), 38.63 ± 2.04 μm (T11-P5), and 43.28 ± 1.59 μm (T23-P2) in tetraploid plants. The average diploid stomatal diameter in the control group was 22.97 ± 1.68 μm. The tetraploid plants T8-P1, T11-P5, and T23-P2 increased to 28.78 ± 1.65 μm, 24.29 ± 2.25 μm, and 25.06 ± 1.92 μm, respectively, but T8-P2 and T8-P3 decreased to 18.96 ± 1.43 μm and 22.27 ± 2.38 μm, respectively. In addition, the average stomatal density of tetraploids was compared to that of the diploid control (151.33 ± 16.53 mm^2^). The stomatal density was significantly lower in T8-P1 (87.07 ± 15.73 mm^2^), T11-P5 (95.36 ± 18.72 mm^2^), and T23-P2 (70.48 ± 10.15 mm^2^), but the density was confirmed to increase in T8-P2 (182.42 ± 33.06 mm^2^) and T8-P3 (178.28 ± 33.06 mm^2^).

The average length and width of the stomata of tetraploid *V. pusanensis* Y.N.Lee (28.87 ± 1.93 μm and 19.85 ± 1.76 μm, respectively) did not show significant differences compared to the control group (28.36 ± 3.51 μm and 19.00 ± 2.29 μm, respectively). However, the density, which was 145.11 ± 12.84 mm^2^ on average in diploid, increased to 190.71 ± 18.72 mm^2^ in the tetraploid.

### 3.5. Phenotypic Traits

To investigate morphological alterations in the colchicine-induced mutant plants of *Veronica*, various leaf parameters were assessed. Phenotypic differences in leaf shape and the presence of epidermal hairs were compared and analyzed.

In diploid plants of *V. nakaiana* Ohwi, the average leaf length, width, and area were 35.35 ± 0.14 mm, 46.75 ± 0.26 mm, and 1661.43 ± 13.54 mm^2^, respectively (Table 4).

**Figure 7:**
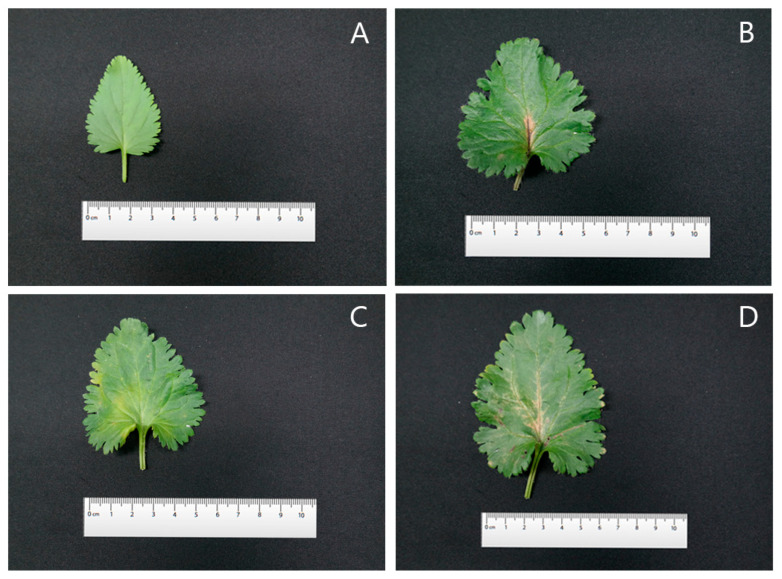
Comparison of leaf size of control and selected mutant plants of *V. nakaiana* Ohwi treated with colchicine: (**A**) control, (**B**) T8-P1 (0.4% + 3 h), (**C**) T8-P2 (0.4% + 3 h), and (**D**) T8-P3 (0.4% + 3 h).

**Figure 8:**
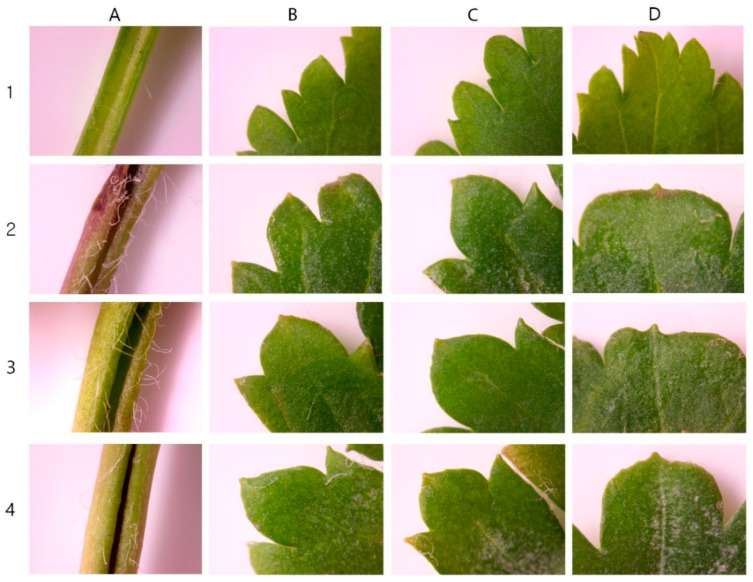
Leaf morphological characteristics of colchicine-induced *V. nakaiana* Ohwi mutants: (**A**–**D**, **left**–**right**) (**A**) petiole; (**B**) leaf margin (**top**); (**C**) leaf margin (**bottom**); and (**D**) leaf apex; (1–4, **top**–**bottom**) 1. control; 2. T8-P1 (0.4% + 3 h); 3. T8-P2 (0.4% + 3 h); and 4. T8-P3 (0.4% + 3 h).

The leaves were cordate in shape with glabrous petioles. The margins were serrated and the leaf tips were acute. Three colchicine-induced mutants of *V. nakaiana* Ohwi—T8-P1, T8-P2, and T8-P3—exhibited significantly altered leaf morphology, including increased leaf length, width, and area (Figure 7 and Figure 8).

For the T8-P1 mutant, the average leaf length, width, and area were 62.30 ± 0.12 mm, 63.75 ± 0.12 mm, and 3971.62 ± 0.17 mm^2^, respectively (Table 4). The leaves maintained a heart-shaped morphology similar to that of diploids, but with dark-purple petioles. Notably, both the number and length of the cilia increased. The margins were sharply serrated and lanceolate and the leaf tips remained acute (Figure 7B and Figure 8B).

T8-P2 exhibited average leaf dimensions of 58.05 ± 0.19 mm in length, 64.23 ± 0.53 mm in width, and 3728.88 ± 21.54 mm^2^ in area (Table 4). The leaves retained a heart-shaped morphology with visibly thickened petioles and a marked increase in the number of epidermal hairs compared to the control. The leaf margins were clearly transformed into a more pronounced serrated pattern, and the leaf tips were acute (Figure 7C and Figure 8C).

T8-P3 demonstrated the largest leaf size among the three mutants, with an average leaf length of 71.99 ± 0.95 mm, width of 90.17 ± 0.50 mm, and area of 6491.88 ± 115.50 mm^2^ (Table 4). The leaves also exhibited a heart-shaped morphology, consistent with the diploid type (Figure 7D). The petioles were thickened and bore sparse ciliates. The margins were sharply serrated, similar to other tetraploid mutants, and the leaf tips were acute (Figure 8D).

In *V. pusanensis* Y.N.Lee, the diploid control plant exhibited an average leaf length of 38.36 ± 0.09 mm, a diameter of 47.90 ± 0.34 mm, and a leaf area of 1837.60 ± 13.04 mm^2^. In contrast, the colchicine-induced polyploid (T11-P4) had a leaf length of 45.30 ± 0.40 mm, diameter of 48.15 ± 0.42 mm, and area of 2181.29 ± 37.00 mm^2^, all of which were greater than those of the diploid control (Table 5).

**Figure 9:**
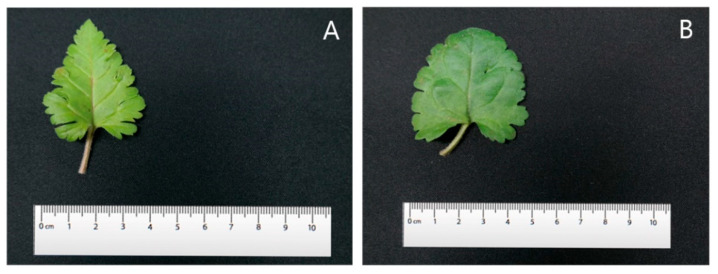
Comparison of leaf size of control and selected mutant plants of *V. pusanensis* Y.N.Lee treated with colchicine: (**A**) control, and (**B**) T11-P4 (0.6% + 1 h).

**Figure 10:**
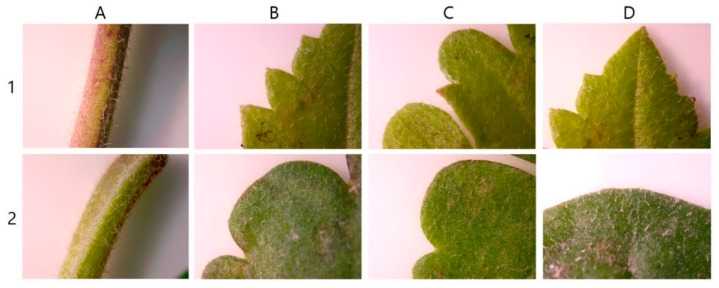
Leaf morphological characteristics of colchicine-induced *V. pusanensis* Y.N.Lee mutant, (1) control; (2) T11-P4 (0.6% + 1 h): (**A**) Petiole; (**B**) leaf margin (**top**); (**C**) leaf margin (**bottom**); and (**D**) leaf apex.

While the diploid leaves were heart-shaped, those of T11-P4 displayed a reniform (kidney-shaped) morphology (Figure 9). The polyploid petioles were thicker than the diploid ones. In terms of leaf margins, the diploid plants exhibited a dentate and serrate pattern, whereas the polyploid plants showed an undulate pattern. The leaf tips were acute in diploid plants but became obtuse in polyploid plants (Figure 10).

## 4. Discussion

Although changing the environmental factors affecting plants can increase their ornamental value, these changes are unstable, and improvements can easily be reversed [28,29,30]. In vitro, which is mainly used in mutation breeding, requires considerable space and time in test tubes and is a complex process in cell culture technology [31]. In addition, studies have shown that both in vitro and in vivo random mutation techniques can be applied with similar results [32]. Polyploid varieties, created by inducing mutations in diploid varieties, have better horticultural characteristics and are often used in breeding programs for economically important plants, thus playing an important role in increasing their commercial value [33,34]. However, polyploidy induction in *Veronica* species using colchicine has not been reported. In this study, we aimed to induce ploidy mutations in native Korean *Veronica* species using colchicine in a simple in vivo environment with basic facilities.

### 4.1. Analysis of Survival and Mutation Rates

Colchicine is a chemical mutagen that induces polyploidy and creates new colors and shapes in horticultural plants [35]. Many previous studies have successfully induced polyploidy in diploid plants using colchicine [36,37]. We evaluated the survival and mutation rates of *Veronica* species according to colchicine treatment concentration and treatment period. The survival rate of *V. nakaiana* Ohwi decreased as colchicine concentration and exposure time increased (Table 1). The toxic effects of colchicine on plants when used at high doses have also been reported in other studies on polyploidy induction [38]. For instance, in *Echeveria* ‘Peerless’, the mortality rate of leaf cuttings increased with higher colchicine concentration [39]. Similarly, Mahna et al. [40] observed that low concentrations of colchicine yielded favorable outcomes, whereas higher concentrations had adverse effects. Interestingly, the survival rate of *V. pusanensis* Y.N.Lee showed little change in survival rate depending on the treatment group, and the ratio was not constant depending on concentration and time (Table 1). This is in contrast with the dose-dependent decreases in germination and survival in colchicine-treated wheat (*Triticum aestivum)* [41]. *Veronica* is relatively resistant to low temperatures and weak to high temperatures [8]. The treatments were conducted during spring for *V. nakaiana* Ohwi and summer for *V. pusanensis* Y.N.Lee, which may be the reason for the inconsistent survival rates of the two *Veronica* species considering the temperature differences. Colchicine produced 27 mutations (6.8%) in *V. nakaiana* Ohwi and 9 mutations (1.5%) in *V. pusanensis* Y.N.Lee, showing a lower mutation rate than *V. nakaiana* Ohwi. However, the mutation rate was higher in both types when the colchicine concentration was lower (Table 1).

### 4.2. Significance of Cytogenetic Findings

Corneillie et al. [42] reported that chemically induced polyploidy is a “high-ploidy syndrome” in which cells expand but grow more slowly than in the control. Therefore, for efficient selection, FCM, a cytogenetic analysis, is widely used in many breeding programs to quickly and accurately estimate the ploidy levels of ornamental plants. Chromosome counting is widely used because of its high reproducibility and reliability [43,44]. In *Echeveria* mutants induced by colchicine, ploidy levels were determined using FCM and chromosome counting [39]. In this study, FCM and chromosome counting were performed on both mutant lines of *Veronica*. The peak in channel 4 (Figure 1 and Figure 2) that indicates tetraploidy (4x), was confirmed by FCM, and 2*n* = 66–68 chromosomes were observed in the diploid 2*n* = 34 (Table 2 and Figure 3 and Figure 4).

### 4.3. Stomatal Characteristics

Stomatal characteristics can also be analyzed to determine plants’ ploidy levels [45,46]. In general, the size of polyploid stomatal cells increases as the ploidy level increases compared to diploid cells, and density decreases [37,47,48]. Compared to the diploid control, the length of the polyploid *V. nakaiana* Ohwi stomatal cells increased, but the width did not change significantly (Table 3 and Figure 5). The stomatal cells of tetraploid *V. pusanensis* Y.N.Lee were also measured to be almost the same size as the diploid cells (Table 3 and Figure 6). Typically, stomatal density decreases as stomatal size increases. However, the results of this study showed that the stomatal density of some *V. nakaiana* Ohwi and *V. pusanensis* Y.N.Lee individuals significantly increased as the stomatal size increased. (Table 3). This is contrast with studies on *Glycyrrhiza glabra* var. *glandulifera*, *Carthamus tinctorius* L., and *Vitis vinifera* L. where a positive correlation was observed between stomata size and ploidy level [49,50]. These conflicting results may be due to the use of FAA fixative to identify stomatal cells. FAA is the most commonly used fixative for plant specimens because of its simplicity and good retention of tissue morphology [51]. All fixatives, including FAA, are toxic. In particular, FAA solutions have a disadvantage in that the combination of fixatives can cause the protoplasm of plant cells to decompose or excessively shrink the specimens [52]. In addition, a previous study on radish, maize, cucumber and wheat showed that when cells were fixed using FAA, they were noticeably shrunk and deformed compared to when methanol and glutaraldehyde were used [53]. The CPD method used to observe stomata is the most commonly used dehydration method for biological sample preparations [54]. However, the leaves of many plants, including the herbaceous plant *Veronica*, are soft, tender, and have high moisture content; thus, problems in the morphology of cells may occur during the dehydration process [53,55]. Alcohol-based solutions such as ethanol and methanol have been proposed as alternatives to the highly toxic FAA fixative solution because they are excellent fixative solutions for protecting the cell surface and plant tissue used before the CPD process [56,57]. Methanol and ethanol, which have low toxicity, can be used instead of the FAA solution to fix *Veronica* cells. It is expected that research on the fixative solution and the processing time of the CPD process will be supplemented during the sample preparation.

### 4.4. Leaf Phenotypic Characteristics

Morphological evaluation is a reliable method for identifying polyploid individuals [23]. Among plant organs, leaves are the most easily observed landmarks for morphological analysis, and the leaves of induced tetraploids can exhibit different characteristics and larger sizes than diploid leaves [58,59]. Compared to the control group, the drained leaves of *V. nakaiana* Ohwi showed a significant increase in length, width, and area, and the overall leaf shape was slightly rounded (Table 4 and Figure 7). The petioles showed an increase in thickness, and the cilia were significantly longer. In addition, the leaf margins were similar to those of the control group, but the leaf apices were horizontal (Figure 8). Although the leaf sizes of the control and polyploid *V. pusanensis* did not differ significantly, the polyploid leaves showed a significant morphological change, with an overall more rounded shape (Table 5 and Figure 9). In particular, the lanceolate shape observed in the control group disappeared in the leaf margin and apex and both showed gently curved shapes (Figure 10). Polyploidization by colchicine has been used to induce the “gigas effect,” a dynamic change in the size of plant organs, and the “bust-up” effect, a new morphology with a variety of color and shape variations in a single species [35,60]. In several studies, polyploid leaves induced using colchicine were larger, thicker, or had different shapes in several ornamental plant species, such as daisy, poinsettia, blue cyanosis, and African violet, and various changes in flower shape and color were observed [61,62,63,64]. Therefore, the main cause of the morphological changes and increase in chromosome number in the treated *Veronica* species could be the effect of colchicine.

## 5. Conclusions

In vitro mutagenesis was performed efficiently in terms of procedure and cost. Polyploid plants were induced using colchicine in native Korean *Veronica* species to confirm changes in leaf size and characteristics. Mutant plants were generated in *Veronica* species when treated with 0.4%, 0.6%, and 1.0% colchicine concentrations for 1 or 3 h. Both FCM and chromosomal analyses confirmed tetraploidy. The size of the stomata was similar or increased; however, a high density was observed owing to the excessive shrinkage caused by the FAA solution. The mutagenesis treatment method and results of this study can be applied to various *Veronica* cultivars to provide new genetic resources, thereby enhancing the genetic diversity available for breeding programs. Future research should include field trials to evaluate the growth stability and ornamental performance of induced mutants under natural conditions. In addition, further optimization of fixation methods for stomatal analysis could improve the accuracy of stomatal evaluations. Broader genetic testing, such as molecular marker analysis or genome-wide screening, is also recommended to verify induced genetic changes and assess their potential for breeding applications.

## Figures and Tables

**Table 1:** Survival and mutation rates (%) of two *Veronica* species induced with colchicine (n = 100).

Code	Treatment	*V. nakaiana* Ohwi		*V. pusanensis* Y.N.Lee	
		Survival Rate (%)	Mutant Rate (%)	Survival Rate (%)	Mutant Rate (%)
T0	Control	40 (40.0)	-	20 (20.0)	-
T1	0.2% + 1 h	30 (30.0)	2 (2.0)	43 (43.0)	1 (1.0)
T2	0.2% + 2 h	19 (19.0)	1 (1.0)	43 (43.0)	1 (1.0)
T3	0.2% + 3 h	29 (29.0)	1 (1.0)	33 (33.0)	1 (1.0)
T4	0.2% + 4 h	25 (25.0)	2 (2.0)	26 (26.0)	0 (0.0)
T5	0.2% + 5 h	14 (14.0)	2 (2.0)	29 (29.0)	1 (1.0)
T6	0.4% + 1 h	29 (29.0)	1 (1.0)	35 (35.0)	2 (2.0)
T7	0.4% + 2 h	26 (26.0)	2 (2.0)	14 (14.0)	0 (0.0)
T8	0.4% + 3 h	19 (19.0)	3 (3.0)	22 (22.0)	0 (0.0)
T9	0.4% + 4 h	18 (18.0)	2 (2.0)	27 (27.0)	0 (0.0)
T10	0.4% + 5 h	15 (15.0)	0 (0.0)	17 (17.0)	0 (0.0)
T11	0.6% + 1 h	16 (16.0)	2 (2.0)	35 (35.0)	2 (2.0)
T12	0.6% + 2 h	11 (11.0)	1 (1.0)	31 (31.0)	0 (0.0)
T13	0.6% + 3 h	15 (15.0)	2 (2.0)	15 (15.0)	0 (0.0)
T14	0.6% + 4 h	11 (11.0)	0 (0.0)	21 (21.0)	0 (0.0)
T15	0.6% + 5 h	11 (11.0)	0 (0.0)	16 (16.0)	0 (0.0)
T16	0.8% + 1 h	14 (14.0)	1 (1.0)	18 (18.0)	0 (0.0)
T17	0.8% + 2 h	14 (14.0)	1 (1.0)	21 (21.0)	0 (0.0)
T18	0.8% + 3 h	9 (9.0)	0 (0.0)	9 (9.0)	0 (0.0)
T19	0.8% + 4 h	14 (14.0)	1 (1.0)	18 (18.0)	0 (0.0)
T20	0.8% + 5 h	6 (6.0)	1 (1.0)	15 (15.0)	0 (0.0)
T21	1.0% + 1 h	10 (10.0)	0 (0.0)	10 (10.0)	0 (0.0)
T22	1.0% + 2 h	16 (16.0)	0 (0.0)	37 (37.0)	0 (0.0)
T23	1.0% + 3 h	7 (7.0)	1 (1.0)	19 (19.0)	1 (1.0)
T24	1.0% + 4 h	14 (14.0)	1 (1.0)	18 (18.0)	0 (0.0)
T25	1.0% + 5 h	5 (5.0)	0 (0.0)	24 (24.0)	0 (0.0)
Total		397 (15.9)	27 (6.8)	596 (23.8)	9 (1.5)

**Table 2:** Chromosome number of *Veronica* mutants treated with colchicine (n = 8).

Colchicine Treatments	Chromosome Number
*V. nakaiana* Ohwi	
Control	2*n* = 34
T8-P1 (0.4% + 3 h)	2*n* = 68
T8-P2 (0.4% + 3 h)	2*n* = 67
T8-P3 (0.4% + 3 h)	2*n* = 68
T11-P5 (0.6% + 1 h)	2*n* = 68
T23-P2 (1.0% + 3 h)	2*n* = 68
*V. pusanensis* Y.N.Lee	
Control	2*n* = 34
T11-P4 (0.6% + 1 h)	2*n* = 66

**Table 3:** Stomata length and density of *Veronica* mutants treated with colchicine (n = 8).

Colchicine Treatments	Length of Stomata (µm)	Diameter of Stomata (µm)	Stomatal Density (mm^2^)
*V. nakaiana* Ohwi			
Control	30.59 ± 1.52 ^x^ a ^y^	22.97 ± 1.68 bc	151.33 ± 16.53 b
T8-P1 (0.4% + 3 h)	46.53 ± 2.90 e	28.78 ± 1.65 e	87.07 ± 15.73 a
T8-P2 (0.4% + 3 h)	34.30 ± 3.88 b	18.96 ± 1.43 a	182.42 ± 33.06 d
T8-P3 (0.4% + 3 h)	36.14 ± 3.12 b	22.27 ± 2.38 b	178.28 ± 33.06 bc
T11-P5 (0.6% + 1 h)	38.63 ± 2.04 c	24.49 ± 2.25 cd	95.36 ± 18.72 a
T23-P2 (1.0% + 3 h)	43.28 ± 1.59 d	25.06 ± 1.92 d	70.48 ± 10.15 a
F-test	**	**	**
*V. pusanensis* Y.N.Lee			
Control	28.36 ± 3.51	19.00 ± 2.29	145.11 ± 12.84
T11-P4 (0.6% + 1 h)	28.87 ± 1.93	19.85 ± 1.76	190.71 ± 18.72
T-test	NS	NS	**

^x^ Mean ± standard error (SE). ^y^ Columns with the same letters are not significantly different by Duncan’s multiple range test at *p* = 0.05. NS, **, nonsignificant or significant at *p* = 0.01, respectively.

**Table 4:** Phenotypic data of colchicine-induced *V. nakaiana* Ohwi (n = 4).

Colchicine Treatments	Leaf Measurement		
	Length (mm)	Width (mm)	Area (mm^2^)
Control	35.53 ± 0.14 ^x^ a ^y^	46.75 ± 0.26 a	1661.43 ± 13.54 a
T8-P1 (0.4% + 3 h)	62.30 ± 0.12 c	63.75 ± 0.12 b	3971.62 ± 0.17 c
T8-P2 (0.4% + 3 h)	58.05 ± 0.19 b	64.23 ± 0.53 b	3728.88 ± 21.54 b
T8-P3 (0.4% + 3 h)	71.99 ± 0.95 d	90.17 ± 0.50 c	6491.88 ± 115.50 d
F-test	**	**	**

^x^ Mean ± standard error (SE). ^y^ Columns with the same letters are not significantly different by Duncan’s multiple range test at *p* = 0.05. **, significant at *p* = 0.01.

**Table 5:** Phenotypic data of colchicine-induced *V. pusanensis* Y.N.Lee (n = 2).

Colchicine Treatments	Leaf Measurement		
	Length (mm)	Width (mm)	Area (mm^2^)
Control	38.36 ± 0.09 ^x^	47.90 ± 0.34	1837.60 ± 13.04
T11-P4 (0.6% + 1 h)	45.30 ± 0.40	48.15 ± 0.42	2181.29 ± 37.00
T-test	**	NS	**

^x^ Mean ± standard error (SE). NS, **, nonsignificant or significant at *p* = 0.01, respectively.

## Data Availability

Data presented in this study are available within the article.

## Abbreviations

The following abbreviations are used in this manuscript:

ANOVA	Analysis of Variance
CPD	Critical Point Drying
DAPI	4’, 6-diamidino-2-phenylindole
DMRT	Duncan’s Multiple Range Test
DNA	Deoxyribonucleic Acid
FAA	Formalin Aceto Alcohol
FCM	Flow Cytometry
*n*	Haploid Genome
PI	Propidium Iodide
rDNA	Ribosomal Deoxyribonucleic Acid
rpm	Revolutions per Minute
RT	Room Temperature
SEM	Single Nucleotide Polymorphism
SSC	Saline Sodium Citrate
